# Prenylated PALM2 Promotes the Migration of Esophageal Squamous Cell Carcinoma Cells Through Activating Ezrin

**DOI:** 10.1016/j.mcpro.2023.100593

**Published:** 2023-06-15

**Authors:** Dan-Xia Deng, Cheng-Yu Li, Zhen-Yuan Zheng, Bing Wen, Lian-Di Liao, Xiao-Jun Zhang, En-Min Li, Li-Yan Xu

**Affiliations:** 1 Guangdong Provincial Key Laboratory of Infectious Diseases and Molecular Immunopathology, Institute of Oncologic Pathology, Shantou University Medical College, Shantou, Guangdong, China; 2 Department of Biochemistry and Molecular Biology, The Key Laboratory of Molecular Biology for High Cancer Incidence Coastal Chaoshan Area, Shantou University Medical College, Shantou, Guangdong, China; 3 Guangdong Esophageal Cancer Research Institute, Shantou Sub-center, Cancer Research Cancer, Shantou University Medical College, Shantou, Guangdong, China; 4 Central Laboratory, Shantou University Medical College, Shantou, Guangdong, China

**Keywords:** prenylation, PALM2, esophageal squamous cell carcinoma, cell migration, ezrin

## Abstract

Proteins containing a CAAX motif at the C-terminus undergo prenylation for localization and activity and include a series of key regulatory proteins, such as RAS superfamily members, heterotrimeric G proteins, nuclear lamina protein, and several protein kinases and phosphatases. However, studies of prenylated proteins in esophageal cancer are limited. Here, through research on large-scale proteomic data of esophageal cancer in our laboratory, we found that paralemmin-2 (PALM2), a potential prenylated protein, was upregulated and associated with poor prognosis in patients. Low-throughput verification showed that the expression of PALM2 in esophageal cancer tissues was higher than that in their paired normal esophageal epithelial tissues, and it was generally expressed in the membrane and cytoplasm of esophageal cancer cells. PALM2 interacted with the two subunits of farnesyl transferase (FTase), FNTA and FNTB. Either the addition of an FTase inhibitor or mutation in the CAAX motif of PALM2 (PALM2^C408S^) impaired its membranous localization and reduced the membrane location of PALM2, indicating PALM2 was prenylated by FTase. Overexpression of PALM2 enhanced the migration of esophageal squamous cell carcinoma cells, whereas PALM2^C408S^ lost this ability. Mechanistically, PALM2 interacted with the N-terminal FERM domain of ezrin of the ezrin/radixin/moesin (ERM) family. Mutagenesis indicated that lysine residues K253/K254/K262/K263 in ezrin’s FERM domain and C408 in PALM2’s CAAX motif were important for PALM2/ezrin interaction and ezrin activation. Knockout of ezrin prevented enhanced cancer cell migration by PALM2 overexpression. PALM2, depending on its prenylation, increased both ezrin membrane localization and phosphorylation of ezrin at Y146. In summary, prenylated PALM2 enhances the migration of cancer cells by activating ezrin.

## Highlights

• Upregulated PALM2 is associated with poor prognosis of esophageal cancer patients.
• PALM2 can be prenylated at the CAAX motif of the C-terminus.
• Prenylated PALM2 activates ezrin to promote the migration of esophageal cancer cells.

## Graphical Abstract

**Figure:**
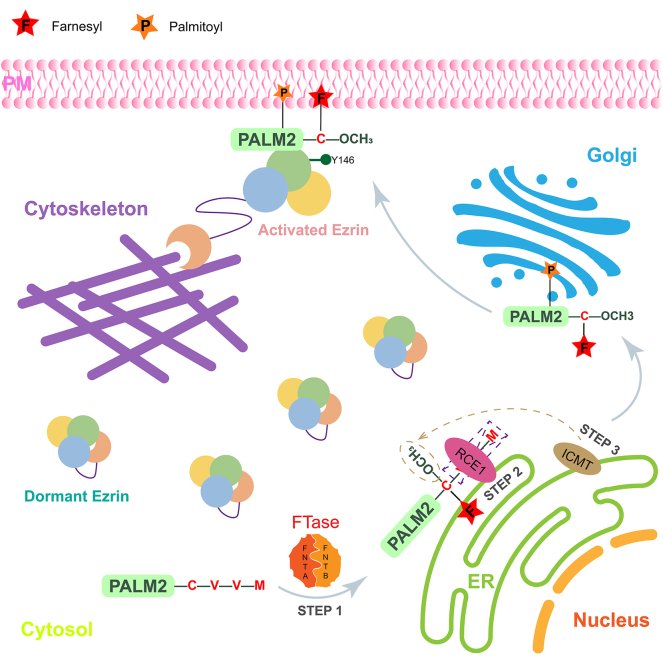

## In Brief

Prenylation is a conservative lipid modification that regulates membrane-protein and protein-protein interactions in cell signaling. Our studies show that the prenylation of PALM2 is catalyzed by FTase, and prenylated PALM2 promotes cell migration of ESCC through activating ezrin by increasing ezrin’s membranous distribution and Y146 phosphorylation. Up-regulated PALM2 is associated with a shorter survival time of esophageal cancer patients and is an independent prognostic factor. This indicates that PALM2 can be a potential target for esophageal cancer prevention and therapy.

Lipidation, a post-translational modification (PTM) of proteins, modulates cellular functions by regulating protein–membrane interactions, protein–protein interactions, protein stability, and enzyme activity (1). At present, the major types of lipidation include prenylation at a C-terminal cysteine residue, myristoylation at an N-terminal glycine residue, palmitoylation at a cysteine residue, and glycosylphosphatidylinositol (GPI) (1, 2). Protein prenylation was first discovered in fungi in 1978 (3), and the first prenylated protein in mammalian cells, farnesylated lamin B, was discovered in 1988 (4, 5). Protein prenylation is an irreversible covalent post-translational modification present in all eukaryotic cells, involving the addition of polyisoprene (also called terpenoid) modification groups, farnesyl, and geranylgeranyl, through thioether bonds on cysteine side chain at the C-terminus of target proteins. More than 2% of proteins are modified by prenylation in humans (1), including a variety of RAS superfamily members, heterotrimeric G proteins, nuclear lamina proteins, and several protein kinases and protein phosphatases (6, 7, 8, 9). These prenylated proteins are involved in intracellular regulatory processes for tumorigenesis, such as division and proliferation, angiogenesis, invasion and metastasis, and apoptosis (10, 11).

Most prenylated proteins contain a CAAX motif at the C-terminus (cysteine for C, any aliphatic amino acid except alanine for A, and any amino acid for X), with the X amino acid residue largely determining which enzyme catalyzes the prenylation modification. Farnesyl transferase (FTase) preferentially prefers alanine, serine, methionine, or glutamine, while leucine, isoleucine, and phenylalanine are favored by geranylgeranyl transferase I (GGTase-type I) (12). Most proteins are catalyzed by only one enzyme, a few proteins such as KRAS are generally farnesylated but can also undergo geranylgeranylation when FTase is inhibited (13, 14), and RHOB can be both farnesylated and geranylgeranylated (15). In addition, some prenylated proteins have a C-terminal CC or CXC structure, which is modified with geranylgeranyl groups catalyzed by geranylgeranyl transferase type II (GGTase-II) (16). In mammals, the pathway for polyisoprene synthesis is the mevalonate pathway (17). After prenylation, proteins require hydrolysis of the AAX residues and carboxymethylation of the exposed cysteine to complete the process of becoming hydrophobic (18).

As representative proteins of prenylation, RAS superfamily proteins play a crucial role in the development of tumors. For instance, the *RAS* gene is one of the most commonly mutated oncogenes in many cancers, and its oncogenic mutations occur in up to 25% of all malignancies (19). If the prenylation of proteins is blocked in tumors, such as mutation of cysteine to serine in the CAAX motif of HRAS, the malignant progression of tumors is hindered (20), and mutation in the CAAX motif of C17orf37 inhibits pseudopodia formation, thereby suppressing tumor invasion and metastasis (21).

Here, paralemmin-2 (PALM2), a predicted prenylated protein, was identified by esophageal cancer proteomics. The most significant feature of *PALM2*, compared with other transcripts, is the last exon. It encodes the CAAX motif of PALM2. We characterized PALM2, an undiscovered protein in esophageal cancer, to examine its prenylated modification and reveal the association between the prenylated PALM2 and the malignant progression of esophageal cancer.

## Experimental Procedures

### Data Sources and Bioinformatic Analysis

**Fig. 1:**
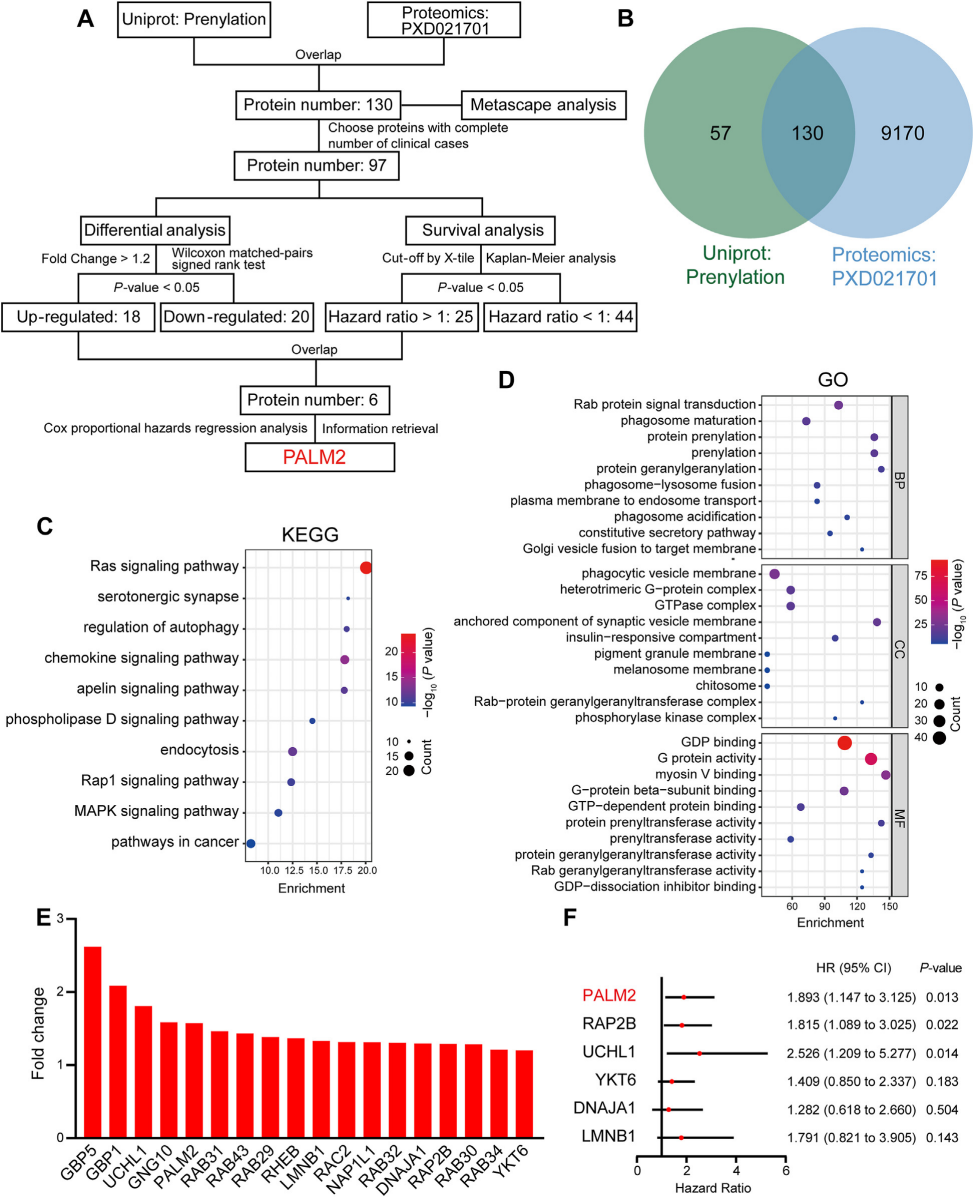
**Bioinformatic analysis of prenylated proteins in esophageal cancer.***A*, flow chart of bioinformatic analysis. *B*, venn diagram of the overlapped proteins between the prenylated proteins in the UniProt database and the esophageal cancer proteomic data (PXD021701). *C* and *D*, bubble charts of some representative KEGG signal pathways and GO gene sets of the overlapped proteins, through Metascape online analysis. *E*, bar chart of the differentially expressed prenylated proteins with FC (tumor *versus* normal tissues) >1.2 and *p* < 0.05 in esophageal cancer. *F*, forest plot of the result of Cox proportional hazards regression analysis of up-regulated prenylated proteins with poor survival in esophageal cancer. CI, confidence interval; FC, fold change; HR, hazard ratio.

Proteomic data on esophageal cancer were generated previously (22). The raw files of the data were obtained from the PRIDE database (accession number PXD021701) (23). The 187 prenylation-related proteins were downloaded from the UniProt database (https://www.uniprot.org/) and overlapped with the proteomic data to obtain the prenylation-related proteins in esophageal cancer. The detailed clinical information and expression of 130 overlapped proteins identified are displayed in supplemental Table S1. Clinical and pathological information is summarized in supplemental Table S2. The 130 proteins were entered into Metascape (https://metascape.org/) (24), which was used to perform both the Kyoto Encyclopedia of Genes and Genomes (KEGG) pathway and Gene Ontology (GO) enrichment analyses. Subsequently, the differential expression analysis (Wilcoxon matched-pairs signed rank test) and survival analysis (Kaplan-Meier analysis, log-rank test, and Cox proportional-hazards regression analysis) of these proteins were computed by SPSS 26.0 software, while a two-tailed *p*-value less than 0.05 was considered to indicate statistical significance. The summarized workflow is illustrated in Figure 1*A*. The results were visualized through the bioinformatics online platform (https://www.bioinformatics.com.cn) and GraphPad Prism 8.0.1.

### Cell Culture and Plasmid Transfection

ESCC cell lines and human embryonic kidney 293 cells transformed with SV40 large T-antigen (HEK 293T) used in this study have been described previously (25, 26). KYSE30 and KYSE200 were grown in Roswell Park Memorial Institute (RPMI) 1640 medium (SH30809.01B, HyClone), while KYSE450 and HEK 293T cells were maintained in Dulbecco’s modified Eagle’s medium (DMEM) (SH30022.01B, HyClone). The growth media contained 10% fetal bovine serum (FBS) (10099-141, Gibco), and 1× penicillin-streptomycin solution (C0178, Maygene), and cells were incubated at 37 °C in a humidified atmosphere containing 5% CO_2_. All cell lines were authenticated by short tandem repeat profiling and were routinely tested for *mycoplasma* contamination.

cDNAs encoding human PALM2, FNTA, and FNTB were cloned from reverse-transcribed cDNAs of ESCC cell lines into a modified pBOBi plasmid vector (25). All plasmids were verified by DNA sequencing. PALM2 truncations and point mutations were amplified by standard PCR cloning from the *PALM2* plasmid. The wild-type, truncations, and point mutations of ezrin plasmids were described before (27). All primers are listed in supplemental Table S3.

For transient expression, EZ trans cell transfection reagent II (AC04L011, Life-iLab) and polyethylenimine (PEI) (23966-2, Polysciences Inc) were used to transfect ESCC and HEK 293T, respectively, according to the manufacturer's instructions. For stable expression, the mixture of three lentivirus packing plasmids, pMDL-gap/polRRE, pVSV-G and pRSC-Rev (1:1:1), were co-transfected with lentiviral vector into HEK 293T cells at a ratio of 1:1. Supernatants containing viral particles were harvested at 48 h and 72 h after transfection and used to infect ESCC cell lines.

For the knockdown of PALM2, siRNA was synthesized by GenePharma, and contained the following sequences: siPALM2-2, 5′-CCAUCAGAAAGGAGUCAAATT-3′; siPALM2-3, 5′-GCCGGAGGCAAACUUGGAUTT-3′. siRNA transfections were performed with jetPRIME *in vitro* DNA & siRNA transfection reagent (114-15, Polyplus).

### Western Blotting

Total proteins of esophageal cancer tissues and their paired normal esophageal epithelial tissues were generated previously (22). The cells were lysed using Laemmli sample buffer (Bio-Rad). Experimental procedures were performed according to standard methods (25). Blots were incubated with primary antibodies, anti-PALM2 (H00114299-M09, Abnova, 1:500), ezrin (E1281, Sigma-Aldrich, 1:1000; MS-661-P1, NeoMarkers, 1:1000), p-ezrin (T567) (3149S, Cell Signaling Technology, 1:1000), p-ezrin (Y146) (sc-12941-R, Santa Cruz, 1:1000), lamin B1 (66095-1-Ig, Proteintech, 1:5000), ATP1A1 (14418-1-AP, Proteintech, 1:10,000), Flag (F3165, Sigma-Aldrich, 1:5000), Myc (2278S, Cell Signaling Technology, 1:1000), HA (sc-7392, Santa Cruz, 1:1000), and GFP (sc-9996, Santa Cruz, 1:1000). Horseradish peroxidase (HRP)-conjugated primary antibodies were GAPDH-HRP (HRP-60004, Proteintech, 1:25,000) and GFP-HRP (HRP-66002, Proteintech, 1:10,000), and HRP-conjugated secondary antibodies were m-IgGκ BP-HRP (sc-516102, Santa Cruz, 1:10,000) and anti-rabbit IgG HRP-linked antibody (7074S, Cell Signaling Technology,1:3000). Signals were detected with enhanced chemiluminescence (ECL) luminol reagent (BL520B, Biosharp) using a ChemiDoc Touch (Bio-Rad). Densitometric analysis was performed with Image Lab software Version 2.0.

### Identification of PALM2 by LC-MS/MS

HEK 293T cells were transfected with Flag-PALM2. Pierce Anti-DYKDDDDK Magnetic Agarose was used to immunoprecipitate Flag-PALM2. IP samples were subjected to SDS-PAGE, and then 1 mm × 1 mm gels sections around 45 kDa and 75 kDa were excised and placed in 300 μl ddH_2_O water for 15 min at RT with agitation. Then 300 μl acetonitrile (ACN) was added and gel slices were incubated for 15 min. The supernatant was discarded and 100 μl ACN was then added for 5 min at RT. Samples were dried in a Speedvac (Eppendorf) and then reduced by mixing with 200 μl of ammonium bicarbonate (100 mM)/DTT (10 mM) and incubation at 56 °C for 30 min. The liquid was removed and 200 μl of ammonium bicarbonate (50 mM)/iodoacetamide (50 mM) was added to the gel slices and incubated at RT in the dark for 30 min. After removal of the supernatant and one 15-min wash with 300 μl ammonium bicarbonate (50 mM), 300 μl ACN was added to dehydrate the gel pieces. The solution was then removed and samples were dried in a Speedvac. For digestion, enough solution of ice-cold trypsin (0.01 μg/μl) in ammonium bicarbonate (20 mM) was added to cover the gel slices and then placed on ice for 30 min. After complete rehydration, the excess trypsin solution was removed, replaced with ammonium bicarbonate (20 mM) to completely cover the gel slices and left overnight at 37 °C. The peptides were extracted twice with 50 μl of 50% ACN/1% formic acid (FA) and vortex-mixing at RT for 30 min. All extracts were pooled and dried in a Speedvac, followed by using ZipTips to purify and concentrate peptides for LC-MS/MS analysis. MS analysis was conducted with an Orbitrap Fusion Lumos (Thermo Fisher Scientific). The peptides were dissolved in 0.1% FA, and separated on an analytical column packed with 2 μm C18 beads (Thermo Fisher Scientific) using a linear gradient ranging from 9% to 28% solvent (80% ACN and 0.1% FA) in 100 min and followed by a linear increase to 45% solvent over 20 min at a flow rate of 300 nl/min. The MS was operated in data-dependent acquisition mode. The spray voltage was set at 2.2 kV and the temperature of the ion transfer capillary was 300 °C. The MS spectra (350–1500 m/z) were collected with 120,000 of resolution, automatic gain control (AGC) of 4 × 10^5^, and 50 ms maximal injection time. Selected ions were sequentially fragmented by higher energy collision dissociation (HCD) with 30% normalized collision energy in a 3 s cycle, specified isolated windows 1.6 m/z, 30,000 of resolution. An AGC of 5 × 10^4^ and 80 ms maximal injection time were used. Dynamic exclusion was set to 15 s.

Data were collected using Xcalibur software (Thermo Fisher Scientific, version 3.0). Raw data were processed using Proteome Discoverer (PD, version 2.4), and MS/MS spectra were searched against the UniProt human proteome database (20,311 entries). The release/download date was August 29th, 2020. Search parameters were as follows: 20 ppm tolerance of precursor mass error, 0.02 Da tolerance of fragment mass error, variable modification for oxidation (Met) (+15.9949 Da), palmitoylation (Lys, Ser, Thr, Cys) (+238.230 Da), acetylation (protein N-terminus) (+42.0106 Da), and carbamidomethylation (Cys) (+57.0215 Da), no fixed modification, and three trypsin missed cleavages allowed. Only peptides of at least six amino acids in length were considered. Peptide and protein identifications were filtered by PD to control the false discovery rate (FDR) < 1%. At least one unique peptide was required for protein identification. Identified proteins are listed in supplemental Table S4.

### Analysis of Nucleus/Cytoplasm Fractionations

Analysis of nucleus/cytoplasm fractionations was performed as described previously (28). KYSE150, KYSE450, and KYSE510 cells were seeded into 6.0 cm dishes. At confluence, cells were washed three times with PBS and lysed using nuclei extraction buffer (NEB) (0.01 M Tris-HCl pH 8.0, 0.01 M NaCl, 0.003 M MgCl_2_, 0.03 M sucrose, 0.5% NP-40) with 1× protease inhibitor cocktail (HY-K0010, MedChemExpress) on ice for 10 min. Cell lysate was centrifuged at 12,500*g* for 5 min at 4 °C next. The supernatant was the cytoplasmic fraction and the precipitate was the nuclear fraction (it was necessary to wash the precipitate thrice or more times using NEB for a clean nuclear fraction).

### Analysis of Cytoplasm/Membrane Fractionations

Analysis of cytoplasm/membrane fractionations was performed as described previously (29). A Minute Plasma Membrane Protein Isolation and Cell Fractionation kit (SM-005, Invent Biotechnologies, Inc) was used to isolate the cytoplasm and membrane proteins of ESCC cells. Experimental procedures were performed according to the operating manual.

### Co-immunoprecipitation

HEK 293T cells or stably transfected KYSE30 cells were seeded into a 10-cm dish. After cell adherence, plasmids were transfected into the HEK 293T cells. After 48 h, cells were washed with PBS and lysed for 10 min on ice with lysis buffer (50 mM Tris-HCl pH 7.5, 120 mM NaCl, 0.5% NP-40), with 1× protease inhibitor cocktail. The cell lysate was incubated with Pierce Anti-DYKDDDDK Magnetic Agarose (A36797, Thermo Fisher Scientific) or Protein A/G Magnetic Beads (HY-K0202, MedChemExpress) coated with anti-GFP (sc-9996, Santa Cruz, 2 μg/ml) at 4 °C overnight. Immunoprecipitates were washed three times with lysis buffer, eluted in SDS sample buffer, boiled for 10 min at 100 °C, and analyzed by Western blotting or mass spectrometry.

### Inhibitor Treatment

FTI 277 (HY-15872A), an FTase inhibitor, GGTI 298 (HY-15871), a GGTase inhibitor (30, 31), and MG132 (HY-13259), a proteasome inhibitor, were purchased from MedChemExpress. 2-Bromohexadecanoic acid (2-BP) (21604-1G), a palmitoyl transferase inhibitor, and CQ (chloroquine) (C6628), a lysosome inhibitor, were purchased from Sigma-Aldrich. KYSE30 and KYSE200 cells stably transfected with Flag-PALM2 were treated with FTI 277 (30 μM) or GGTI 298 (10 μM) for 48 h, 2-BP for 24 h, MG132 (20 μM) and CQ (40 μM) for 6 h, and then, the cells were used for Western blotting or immunofluorescence analysis.

### Immunofluorescence

KYSE30 and KYSE200 cells were seeded onto coverslips (801010, NEST) in 12-well plates. After treatment or transfection, the cells were fixed with methanol (−20 °C) for 10 to 15 min. The cells were washed three times with PBS, and blocked in 5% normal donkey serum (017-000-121, Jackson) for 1 h at 25 °C. The cells were incubated with anti-Flag (F3165, Sigma-Aldrich, 1:1000), and ATP1A1 (14418-1-AP, Proteintech, 1:100) overnight and stained with Alexa Fluor 488-conjugated AffiniPure donkey anti-mouse IgG (H + L) (1:200, 715-545-150, Jackson) or Alexa Fluor 594-conjugated AffiniPure donkey anti-mouse IgG (H + L) (1:200, 711-585-152, Jackson) secondary antibodies for 1 h at 25 °C. Then, the cells were washed three times with PBS and counterstained with 4′,6-diamidino-2-phenylindole dihydrochloride (DAPI) (D9564, Sigma, 1 μg/μl) at 25 °C for 10 min to visualize the nuclei. Images were obtained and processed by laser-scanning confocal microscopy (LSM800, Carl Zeiss).

### Analysis of Detergent-Soluble and -Insoluble Fractions

Analysis of detergent-soluble and -insoluble fractions was performed as described previously (27, 32). In KYSE30 and KYSE200 cells, cellular fractions were obtained from confluent cultures in 6-well plates. Soluble fractions were prepared by a 1 min extraction with 0.5% Triton X-100 buffer (50 mM MES, 3 mM EGTA, 5 mM MgCl_2_, 0.5% Triton X-100, pH 6.4) at 20 °C and supplemented with 5× SDS sample buffer. The insoluble fractions were extracted with SDS sample buffer at 75 °C. Samples were analyzed by Western blotting.

### qRT-PCR

Total RNA was extracted through TRIzol (15596018, Life) and trichloromethane. Reverse transcription was performed with HiScript III RT SuperMix for qPCR (+gDNA wiper) (R323-01, Vazyme). qRT-PCR procedure was performed with 2× ChamQ Universal SYBR qPCR Master Mix (Q711-02, Vazyme) and ABI 7500 PCR instrument. The primers for β-actin were 5′-CAACTGGGACGACATGGAGAAA-3′ (forward) and 5′-GATAGCAACGTACATGGCTGGG-3′ (reverse). The primers for *PALM2* were 5′-CACGAACAGCAGAACCATCAC-3′ (forward) and 5′-CTCTGGGCCAATGGTAGATGT-3′ (reverse).

### Migration Assays

Cell migration was evaluated using wound-healing and transwell assays, which were performed as described previously (27). Briefly, KYSE30 and KYSE200 cells were transfected with Flag-vector, Flag-PALM2 or Flag-PALM2^C408S^. KYSE150 and KYSE510 were transfected with siRNA. After 36 h, cells were starved in serum-free medium for 12 h. In the wound-healing assay, a scratch was made across the monolayer using a sterile pipette tip. Wound closure was imaged at 0 and 12 to 48 h with a microscope (magnification, ×100). For the transwell assay, 5 × 10^4^ cells were added into the upper transwell chamber (353097, BD Biosciences) in serum-free medium, while RPMI 1640 medium with 10% fetal bovine serum was added to the lower chamber. The chambers were incubated for 24 h or 48 h at 37 °C. The cells that had traversed the membrane to the lower side were fixed with methanol and then stained with 0.1% crystal violet at room temperature for 30 min. Cells in the lower side of the membrane were then counted under a microscope (magnification, ×200). Fiji, an image processing package Image J, was used to measure wound-healing area and the number of cells on the lower surface of the transwell membrane.

### Identification of PALM2-Interacting Proteins by LC-MS/MS

HEK 293T cells were transfected with Flag-PALM2, and Pierce Anti-DYKDDDDK Magnetic Agarose was used for immunoprecipitation. IP samples were subjected to SDS-PAGE and then the gel slice containing proteins was cut off. Decolorization and dehydration with ACN were used to treat the gel. Dithiothreitol (10 mM) was added for 1 h at 37 °C, followed by incubating with iodoacetamide (20 mM) for 40 min at 25 °C in the dark, precipitated with 25% trichloroacetic acid, and washed twice with precooled acetone (−20 °C). Ammonium acetate (100 mM) was used to resuspend the precipitate. First, protein digestion was performed at 37 °C overnight using trypsin at a mass ratio of 50:1 (protein: trypsin). Second, digestion at a ratio of 100:1 was performed at 37 °C for 6 h, then 10% trifluoroacetic acid was added to terminate the enzymatic digestion. The sample was desalted using a C18 solid-phase extraction column and dried *via* vacuum centrifugation. For peptide separation, 50 μl 80% ACN containing 0.1% FA was used to elute the peptides. Peptides were loaded onto an anion-exchange PolyWAX LP column (PolyLC) in a Dionex Ultimate 3000 UPLC system (Thermo Scientific). The mobile phases were comprised of solvent A (90% ACN, 0.1% FA) and solvent B (30% ACN, 0.1% FA). The peptides were separated into 60 fractions with a 60 min gradient at a flow rate of 1 ml/min, then were pooled into 12 fractions and dried *via* vacuum centrifugation. The peptides were dissolved in 0.1% FA and loaded onto an Acclaim PepMap 100 C18 Trap column (Thermo Scientific), and then separated on a New Objective PicoChip column with a 5 to 35% solvent B gradient over 90 min, 35 to 50% solvent B over 15 min, 50 to 90% solvent B over 5 min, and 100% solvent B for 10 min, at a flow rate of 300 nl/min in an Orbitrap Elite mass spectrometer (Thermo Scientific) coupled with an EASY-nLC 1000 nanoflow LC system (Thermo Scientific). A data acquisition procedure alternated between MS full-scan (range: 300–1800 m/z, resolution: 120,000 at 200 m/z, AGC: 1 × 10^6^) and MS/MS scans (resolution: 60,000, AGC: 2 × 10^5^, maximum injection time: 200 ms, HCD: 35%, dynamic exclusion time: 30 s).

Data were collected using Xcalibur software (Thermo Fisher Scientific, version 3.0). The acquired data were searched based on the Uniprot database of *Homo sapiens* proteome (protein number: 79052; canonical format; released on 30.10.2021) using Proteome Discoverer 2.2. Precursor mass tolerance was set to 10 ppm and a fragment match tolerance of 0.5 Da. Trypsin was used as the protein-cleaving enzyme, and a maximum of two missed cleavages were accepted. Variable modifications were Oxidation (M), Acetyl (protein N-terminal), and Carbamidomethylation (C) was specified as fixed modifications. A global FDR was less than 1%. Identified interacting proteins are listed in supplemental Table S4.

### CRISPR/Cas9 Knockout

Generation of knockout cell lines by using CRISPR/Cas9 technology was carried out by transfection with pCMV-Cas9 BSD and pcDNA3.3-gRNA (25). The gRNA sequence was designed with https://zlab.bio/guide-design-resources. We chose the second exon of *EZR* to predict the gRNA sequence, 5′-CAATCCAGCCAAATACAAC-3′. Plasmids were transfected into KYSE450 cells using Lipofectamine 3000 transfection reagent. Two days after transfection, blasticidin (R21001, Thermo Fisher) was added to the culture medium, and the cells were cultured for another 2 to 3 days. Single cells were seeded into 96-well plates for 15 days or more, depending on the cell growth rate. Western blotting and DNA sequencing were used to screen for EZR-deficient clones.

### Experimental Design and Statistical Rationale

This study details results obtained from HEK 293T cells overexpressing PALM2. According to different aims, the separating range of samples in SDS-PAGE and Coomassie brilliant blue staining were selective. We qualitatively analyzed the total proteins captured (trypsin digest) as well as peptides eluted. Strict biological replications were not taken in this research, but there were mutual verifications between our two mass spectrometry experiments.

## Results

### PALM2 Is an Upregulated Prenylated Protein Associated With Poor Prognosis in Esophageal Cancer

To explore prenylated modified proteins in esophageal cancer, we first downloaded the prenylation-related protein set from the UniProt protein database, containing 187 reviewed proteins. 130 of 187 proteins were detected in the published proteomic data of esophageal cancer in our laboratory (22) (Fig. 1*B*). Based on this, these 130 proteins were entered into Metascape to analyze which functions and pathways these prenylation-related proteins were mainly engaged. KEGG enrichment analysis showed that prenylation-related proteins detected in esophageal cancer were mainly engaged in the RAS signaling pathway, serotonin synapse, regulation of autophagy, chemokine signaling pathway, and apelin signaling pathway (Fig. 1*C*). GO enrichment results showed that enriched biological processes were Rab protein signal transduction, plasma membrane trafficking to nucleosomes, constitutive secretory pathway, phagosome acidification, and Golgi vesicle fusion to target membrane. Enriched cellular components were phagocytic vesicle membrane, heterotrimeric G protein complex, pigment granule membrane, melanosome membrane, chitosome, and phosphatase kinase complex. Enriched molecular functions included GDP binding, G protein activity, myosin V binding, GTP-dependent protein binding, prenyl transferase activity, geranylgeranyl transferase activity, G-protein beta subunit binding, and GDP dissociation inhibition (Fig. 1*D*). In summary, enrichment analyses showed that prenylated proteins in esophageal cancer existed in the membranes of various cellular components and were involved in material transport, membrane fusion, and activation of signal transduction, and focused on the functions of small G proteins, such as GDP/GTP conversion.

Next, we researched prenylated proteins that impacted the survival of patients with esophageal cancer. First, in order to facilitate the analysis and design, prenylated proteins, that were undetected in all 124 esophageal cancer patients, were eliminated. After that, differential protein expression and Kaplan-Meier analysis were used to screen up-regulated proteins related to poor prognosis in esophageal cancer. There were 18 proteins with fold change (FC) larger than 1.2 (Fig. 1*E*) and 25 proteins associated with poor prognosis (Table 1). Combined with the results of the above analysis, it was found that 6 prenylated proteins were upregulated and were associated with poor prognosis in esophageal cancer (bold font in Table 1). Cox proportional hazards regression analysis of these 6 proteins revealed three independent prognostic risk factors, PALM2, RAP2B and UCHL1 (Fig. 1*F*). RAP2B and UCHL1 have been studied for their structure, localization, and functions, and their prenylation modifications have also been confirmed and can affect their functions (33, 34, 35, 36, 37, 38, 39, 40, 41). Consequently, PALM2, which had the CAAX motif at the carboxyl terminus but had unclear cellular localization and molecular function, was chosen for further study.

**Table 1:** Kaplan–Meier analysis (HR >1) of prenylated proteins in esophageal cancer

Protein symbol	Low expression No.	High expression No.	Overall survival *p* value^a^
BROX	111	13	0.015
**DNAJA1**	110	14	0.042
DNAJB2	30	94	0.026
EHBP1	93	31	0.001
GNG2	114	10	0.014
HRAS	104	20	0.022
**LMNB1**	114	10	0.011
**PALM2**	94	30	0.007
PEX19	110	14	0.039
RAB13	113	11	0.010
RAB1A	31	93	0.026
RAB28	87	37	0.049
RAB2A	109	15	0.009
RAB4B	14	110	0.016
RAB6A	103	21	0.004
RABL3	113	11	0.004
RALA	90	34	0.001
RALB	112	12	0.002
**RAP2B**	88	36	0.018
RAP2C	88	36	0.006
RHOA	101	23	0.003
RHOG	105	19	0.016
**UCHL1**	111	13	0.001
**YKT6**	91	33	0.019
ZFAND2B	103	21	0.014

a Log-rank test of Kaplan-Meier analysis; *p* < 0.05 was considered significant.

### Expression and Localization of PALM2 in Esophageal Cancer

**Fig. 2:**
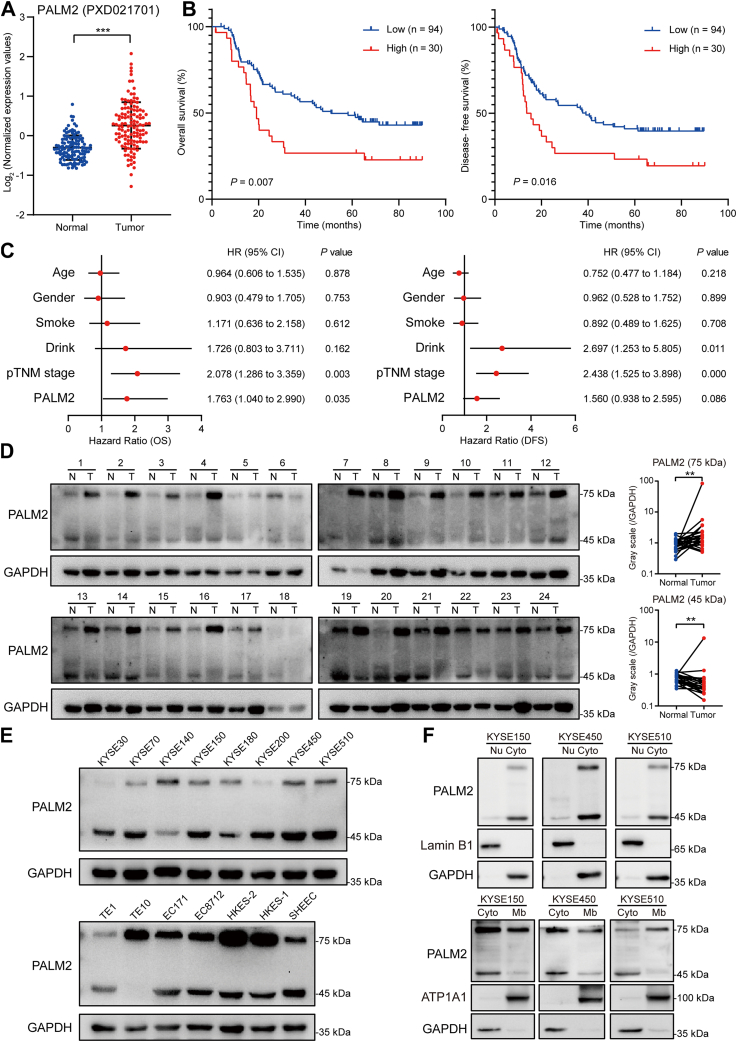
**Expression and clinical significance of PALM2 in esophageal cancer.***A*, scatter plot of PALM2’s relative expression in esophageal tumor and matched normal tissues (data source: PXD021701), Wilcoxon matched-pairs signed rank test, ∗∗∗*p* < 0.001. *B*, Kaplan-Meier survival curve of PALM2 expression for overall survival time and disease free survival time of esophageal cancer patients (data source: PXD021701). *C*, forest plot of the result of Cox proportional hazards regression analysis of PALM2 and clinical information of esophageal cancer patients (data source: PXD021701). *D*, Western blotting analysis of PALM2 expression in ESCC tissues, with GAPDH as the loading control. *Right*, *gray* value analysis of the *left*, Wilcoxon matched-pairs signed rank test, ∗∗*p* < 0.01. *E*, Western blotting analysis of PALM2 expression in ESCC cell lines. *F*, PALM2 is mainly located in the cytoplasm and membrane. Isolation of the different components of ESCC is describred in Experimental procedures, and then Western blotted and probed for the expression of PALM2, with GAPDH as the cytoplasmic internal reference, lamin B1 as the nuclear internal reference, and ATP1A1 as the membrane internal reference. CI, confidence interval; HR, hazard ratio; pTNM, pathologic Tumor-Node-Metastasis.

According to the proteomic data, it can be concluded that the expression of PALM2 is up-regulated in esophageal cancer (Fig. 2*A*), and high expression was associated with shorter survival time of patients with esophageal cancer (Fig. 2*B*), which was not affected by the age, sex, smoking, drinking, and other factors of patients with esophageal cancer, and was an independent prognostic factor for esophageal cancer patients (Fig. 2*C*).

To validate the results from the bioinformatic analysis, Western blotting was performed on 24 pairs of esophageal cancer tissues and their matched normal esophageal epithelial tissues (Fig. 2*D*). The predicted molecular weight of PALM2 is about 45 kDa. In addition, it was also found that there was a significant band at 75 kDa. The two bands that were identified both were PALM2 by LC-MS/MS (supplemental Fig. S1). After quantitative analysis of gray values, the Wilcoxon signed-rank test was used to assess its statistical significance. The relative expression level of the 75 kDa protein was higher in most cancer tissues than that of normal tissues, while the trend of the 45 kDa protein was the opposite. Combining this data with the bioinformatic analysis suggests that the 75 kDa PALM2 may be the functional form in esophageal cancer. We also examined PALM2 expression in ESCC cell lines. Among them, the 75 kDa PALM2 expression was lower in KYSE30 and KYSE200 cells (Fig. 2*E*). Cell fractionation and Western blotting were used to investigate the location of PALM2. KYSE150, KYSE450, and KYSE510 cells were selected for the analysis of nucleus/cytoplasm and cytoplasm/membrane distributions. As shown in Figure 2*F*, PALM2 was mainly localized in the cytoplasm and plasma membrane.

### PALM2 Is Modified by Prenylation Catalyzed by FTase and Palmitoylation

**Fig. 3:**
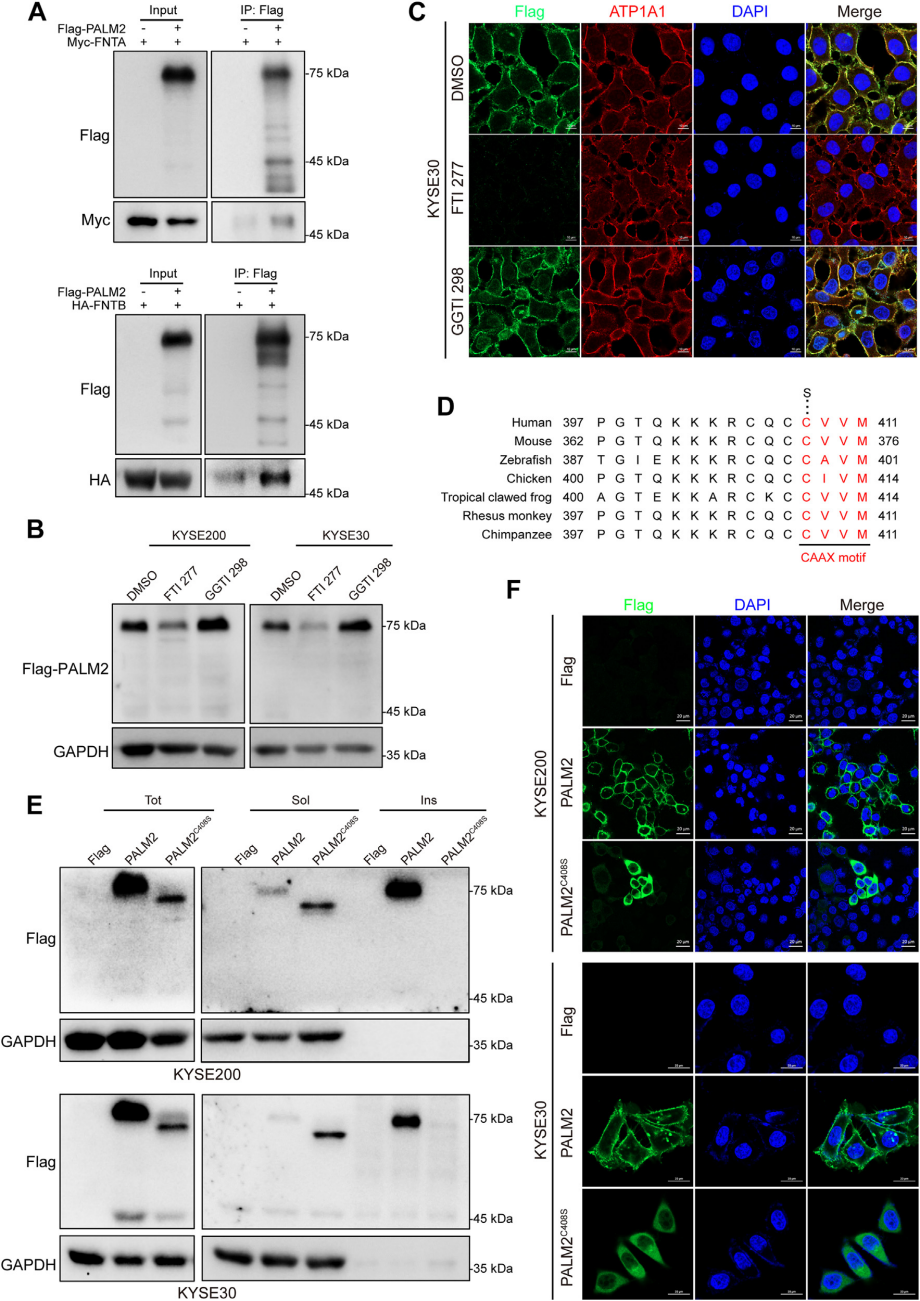
**PALM2 prenylation is catalyzed by FTase.***A*, PALM2 interaction with Ftase subunits FNTA and FNTB. HEK 293T cells were transfected with Flag-PALM2 and Myc-FNTA or HA-FNTB. Following Flag IP, samples were analyzed by Western blotting with antibodies against Flag and Myc or HA. *B* and *C*, effect of ftase inhibitor on prenylated PALM2. Cells stably transfected with Flag-PALM2 were treated with FTI 277 (30 μM), an FTase inhibitor, or GGTI 298 (10 μM), a GGTase inhibitor, for 48 h, after which Western blotting and IF were used to detect the expression of PALM2, scale bar = 10 μm. *D*, C-terminal sequence of PALM2 in several species. Mutant human PALM2 (C408S) was constructed. *E*, PALM2 was mostly insoluble, but PALM2^C408S^ was soluble. Cells were transfected with Flag-PALM2 or PALM2^C408S^ and then the total, soluble, and insoluble proteins were extracted to detect the expression *via* antibody against Flag. *F*, PALM2 is predominantly located in the membrane and PALM2^C408S^ in the cytoplasm. Cells were stably transfected with PALM2 or PALM2^C408S^ and then IF was used to display the location *via* antibody against Flag, scale bar = 20 μm.

The carboxyl-terminal amino acid residues of PALM2 are CVVM, and the last amino acid, methionine, is usually recognized by FTase. Thus, plasmids encoding FNTA and FNTB, two subunits of FTase, were co-transfected with PALM2 into HEK 293T cells. Co-IP experiments were performed and showed PALM2 interacted with both FNTA and FNTB (Fig. 3*A*). In addition, the inhibitor FTI 277 or GGTI 298 was added into the medium to verify which transferase catalyzed the prenylation of PALM2. As would be expected if FTase prenylated PAML2, FTI 277 suppressed the membranous location of PALM2, but GGTI 298 did not (Fig. 3, *B* and *C*). In addition, FTI 277 decreased the protein stability of PALM2 (supplemental Fig. S2). S-palmitoylation is always coupled with prenylation to increase its lipophilicity. PALM2 should be palmitoylated, based on predicted palmitoylation sites (supplemental Fig. S3*A*) and MS analysis (supplemental Fig. S3*B*), and the palmitoylation site is specific for the 75 kDa form rather than the 45 kDa form. Some predictions show that PALM2 has many potential PTMs (supplemental Fig. S4). After treatment with 2-BP, a palmitoyl transferase inhibitor, PALM2’s distribution in the soluble fraction was increased, and decreased in the insoluble fraction (supplemental Fig. S3*C*). In contrast to the inhibition of farnesylation, inhibition of palmitoylation did not cause PALM2 instability.

The CAAX motif of PALM2 is conservative. There are many species with the motif in the carboxyl terminus (Fig. 3*D*). Mutation of the cysteine should inhibit the prenylation of proteins (42). Therefore, a plasmid encoding a cysteine-to-serine mutation at the carboxyl terminus of PALM2 (PALM2^C408S^) was constructed. Surprisingly, transfection of PALM2^C408S^ into KYSE30 and KYSE200 cells revealed that PALM2^C408S^ had a smaller molecular weight and different distribution compared to PALM2. (Fig. 3, *E* and *F*). Taken together, these results support that PALM2 is prenylated in a reaction catalyzed by FTase.

### PALM2-Enhanced Cell Migration of ESCC Cells is Dependent on Prenylation

**Fig. 4:**
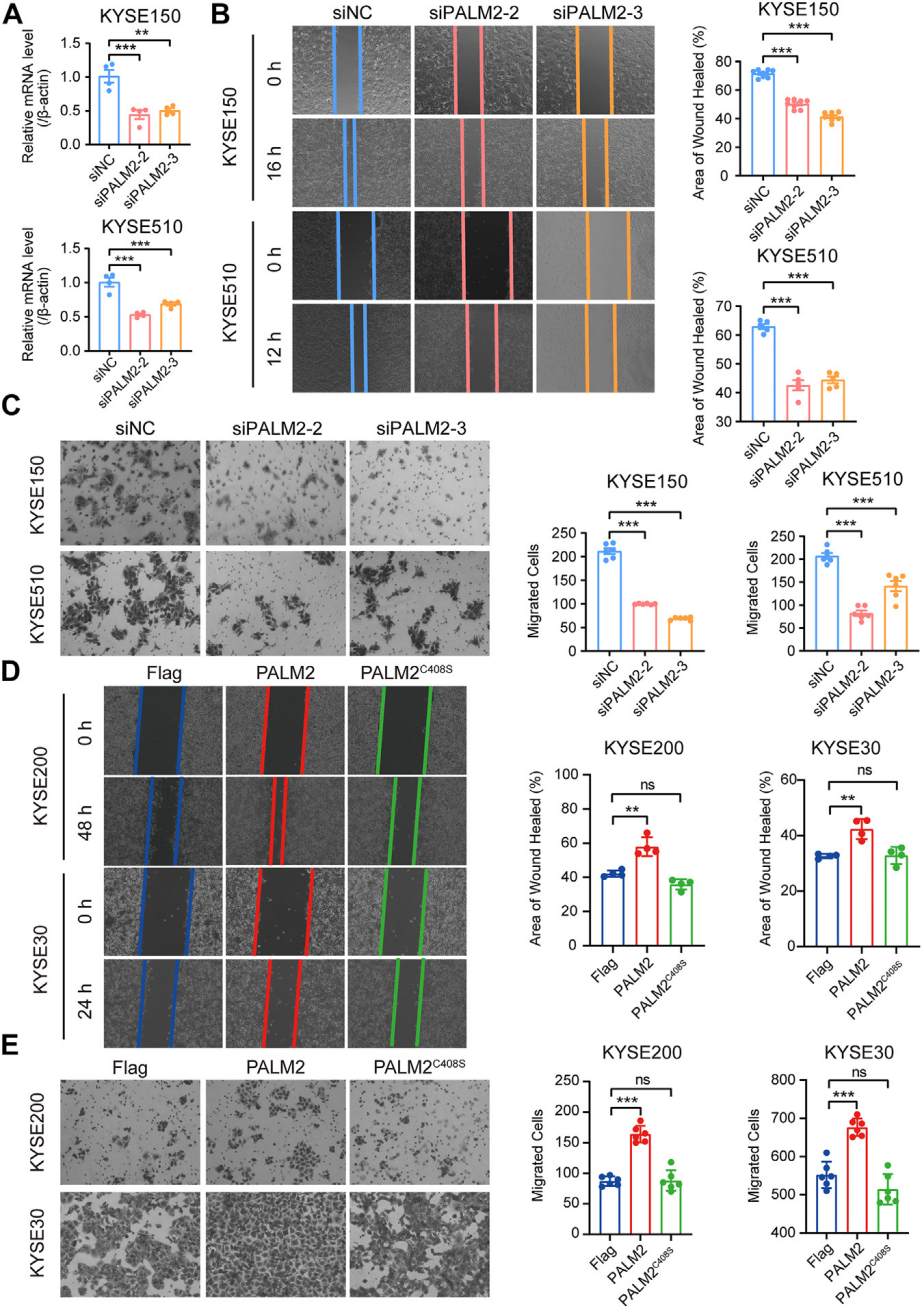
**PALM2-enhanced migration of ESCC is dependent on PALM2 prenylation.***A*, knockdown of PALM2 *via* siRNA in KYSE150 and KYSE510. *B* and *C*, wound healing and transwell assays showing that PALM2 knockdown reduced cell migration in KYSE150 and KYSE510. *D* and *E*, wound healing and transwell assays showing that PALM2, rather than PALM2^C408S^, enhanced cell migration in KYSE200 and KYSE30 cells. All the data are presented as both individual values and the mean ± SD, and determined by one-way ANOVA, ∗∗*p* < 0.01, ∗∗∗*p* < 0.001.

PALM2 is prenylated and targeted to the plasma membrane, and is positively correlated with lymph node metastasis of ESCC patients (Table 2), suggesting that the function of PALM2 may be related to invasion and metastasis. Wound healing and transwell assays showed that PALM2 knockdown in KYSE150 and KYSE510 inhibited cell migration (Fig. 4*A*–*C*). Furthermore, we overexpressed Flag-PALM2 or Flag-PALM2^C408S^ in KYSE30 and KYSE200 cells for wound healing and transwell migration assays. The wound healing rate of KYSE30 and KYSE200 cells transfected with PALM2 was higher than that of the control group and PALM2^C408S^ mutation group (Fig. 4*D*). Transwell migration assays also confirmed the results (Fig. 4*E*). At this point, we conclude that PALM2 can promote the migration of esophageal cancer cells and is dependent on its prenylation.

**Table 2:** Correlation between PALM2 and clinicopathological characteristics in the proteomic data of esophageal cancer

Variables	PALM2		*p*-value^a^
	Low	High	
Age (years)			
≤58	45	17	0.402
>58	49	13	
Gender			
Male	76	21	0.210
Female	18	9	
Smoke			
Yes	34	8	0.338
No	60	22	
Drink			
Yes	9	5	0.285
No	85	25	
Tumor location			
Upper	4	2	0.311
Middle	54	21	
Lower	36	7	
Histological grade			
G1	18	5	0.709
G2	63	19	
G3	13	6	
Primary tumor			
T1+T2	17	3	0.445
T3+T4	77	27	
Regional lymph node			
N0	50	11	0.000
N1	28	6	
N2	9	13	
N3	7	0	
pTNM stage			
I+II	50	11	0.115
III+IV	44	19	

a Pearson chi-square, continuity correction or likelihood ratio test; *p* < 0.05 was considered significant.

### PALM2 Interaction With Ezrin is Dependent on Its Prenylation

**Fig. 5:**
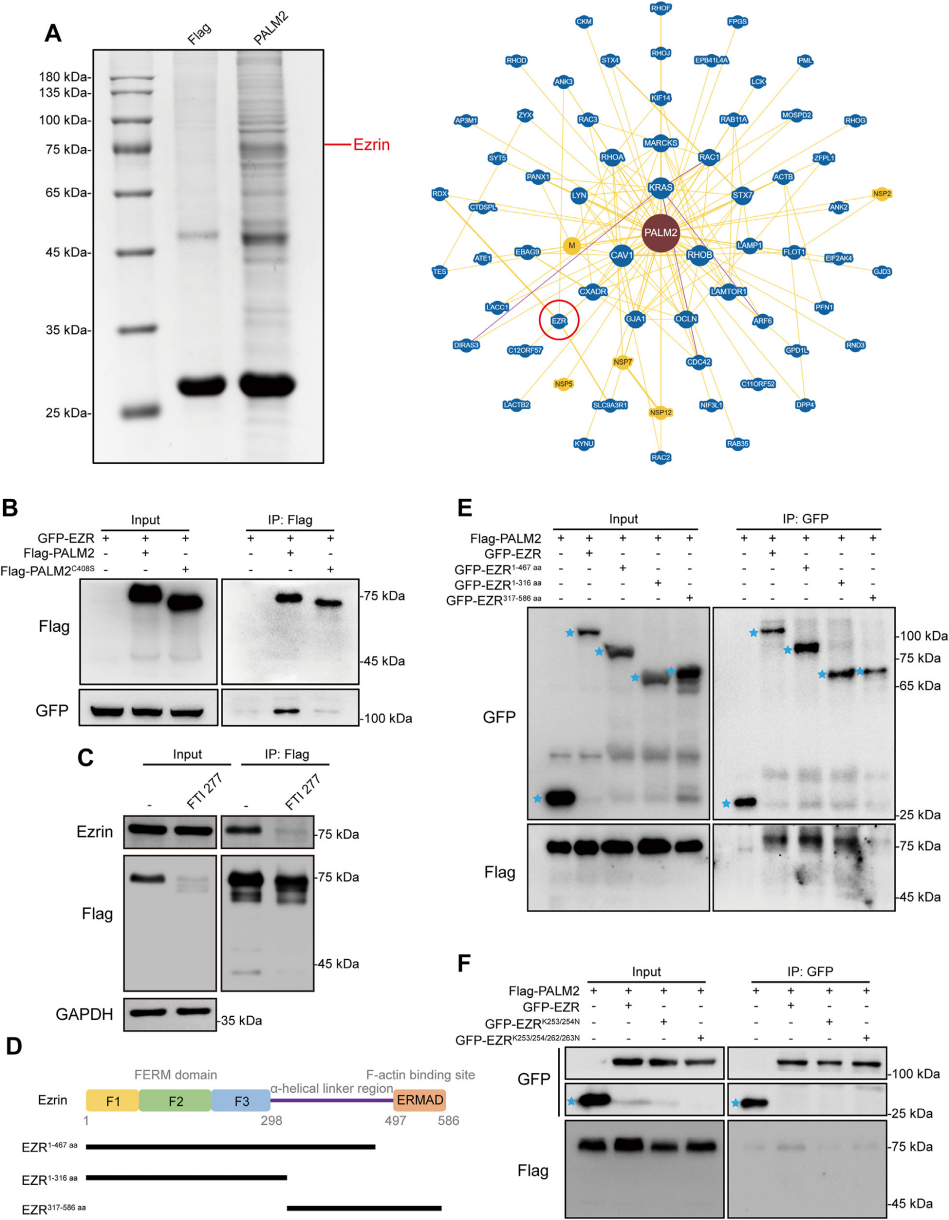
**PALM2 interacts with ezrin.***A*, *left*, Coomassie blue staining of the IP samples before the mass spectrometry analysis; *right*, interacting-protein network of PALM2 in the BioGRID database. *B*, PALM2 interacted with ezrin, but PALM2^C408S^ did not. HEK 293T cells were co-transfected with GFP-EZR and Flag-PALM2 or PALM2^C408S^ and following Flag IP, samples were characterized by Western blotting with antibodies against Flag and GFP. *C*, FTI 277, FTase inhibitor, suppressed the interaction between PALM2 and ezrin. After HEK 293T cells were transfected with Flag-PALM2 for 12 h, FTI 277 was added for 48 h, and following Flag IP, samples were characterized by Western blotting with antibodies against Flag and ezrin. *D*, sketch map of ezrin and its truncations. *E*, PALM2 interacted with the FERM domain of ezrin. Cells were co-transfected with Flag-PALM2, and the different truncated types of ezrin, and then GFP IP samples were characterized by Western blotting with antibodies against Flag and GFP. Blue stars indicate GFP-vector, GFP-EZR, GFP-EZR^1-467 aa^, GFP-EZR^1-316 aa^, and GFP-EZR^317-586 aa^ proteins. *F*, PALM2 interacts with K253/254/262/263 in the ezrin FERM domain. Cells were co-transfected with Flag-PALM2, and the mutant ezrins, and then GFP IP samples were characterized by Western blotting with antibodies against Flag and GFP. *Blue stars* indicate GFP vector.

We further pursued PALM2-interacting proteins through IP-MS and identified ezrin of the ezrin/radixin/moesin (ERM) family, which regulates cell migration by connecting the cytoskeleton to the cell membrane (43), in contrast with the BioGRID database (https://thebiogrid.org/) (Fig. 5*A*). To confirm the interaction between PALM2 and ezrin, co-transfection of GFP-ezrin and either Flag-PALM2 or Flag-PALM2^C408S^ into HEK 293T cells showed that the interaction of PALM2 and ezrin was dependent on PALM2 prenylation (Fig. 5*B*). Similar results were obtained with KYSE30 cells (supplemental Fig. S5*A*). Moreover, co-immunoprecipitation of PALM2, following co-transfection GFP-ezrin and Flag-PALM2^ΔCAAX^ (the truncated type) into HEK 293T cells, showed a lack of association of ezrin with Flag-PALM2^ΔCAAX^ (supplemental Fig. S5*B*), and FTI 277, FTase inhibitor, could also disrupt the interaction of PALM2 with ezrin (Fig. 5*C*). For pursuing the interaction between PALM2 and ezrin, different truncated forms of ezrin were designed (structures shown in Fig. 5*D*). Immunoprecipitation viewed that PALM2 interacted with the N-terminal FERM domain of ezrin (Fig. 5*E*). The lysine residues K253/254 and K262/263 in the FERM domain are key sites through which ezrin attaches to the plasma membrane (32). Mutant ezrin K253/254/262/263N had reduced the interaction between ezrin and PALM2 (Fig. 5*F*). The above results suggested that PALM2 interaction with ezrin depends on PALM2 prenylation and ezrin association with the cell membrane.

### Ezrin Is Essential for PALM2-Mediated Enhancement of ESCC Cell Migration

**Fig. 6:**
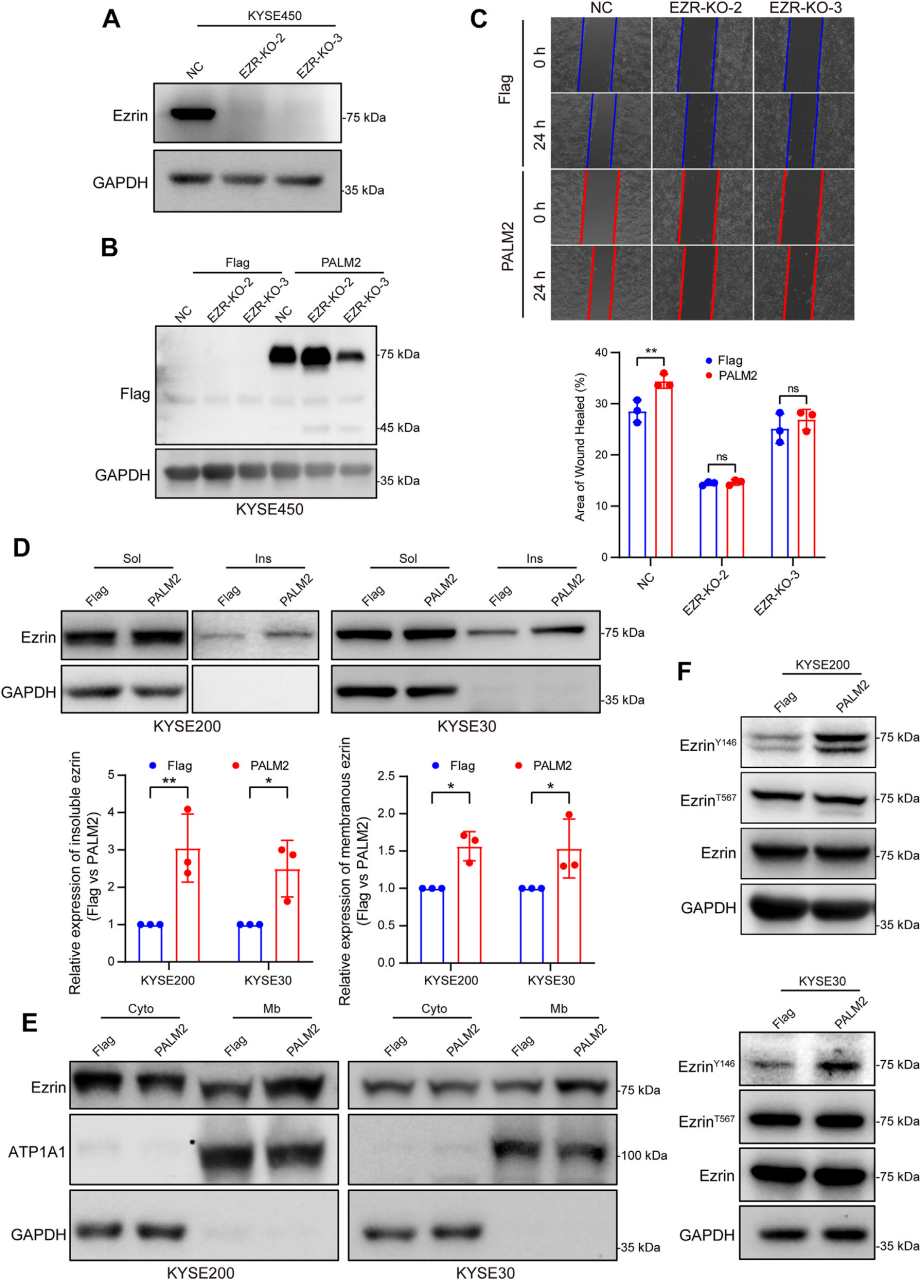
**PALM2 increases ezrin membrane localization and Y146 phosphorylation to activate ezrin.***A*, Western blotting analysis of ezrin knockout in KYSE450 cells by CRISPR/Cas9 technology. *B*, Western blotting analysis of the Flag-PALM2 transfection into the control group (NC) and ezrin-KO groups (Ezrin-KO-2 and Ezrin-KO-3) of KYSE450 cells. *C*, PALM2 promotes cell migration through ezrin. Ezrin-KO cells were transiently transfected with Flag-PALM2 and a wound-healing assay was used to determine cell migration. All data are presented as both individual values and the mean ± SD, and determined by two-way ANOVA analysis, ∗∗*p* < 0.01. *D* and *E*, Western blotting analysis of the distribution of ezrin by isolating detergent-soluble and -insoluble, and cytoplasm/membrane fractions of cells stably transfected with Flag-PALM2. The method of separation is described in the Experimental procedures. All data are presented as both individual values and the mean ± SD and determined by two-way ANOVA, ∗*p* < 0.05, ∗∗*p* < 0.01. *F*, Western blotting analysis of ezrin, ezrin^Y146^, and ezrin^T567^ expression in cells stably transfected with Flag-PALM2.

To explore the effect of ezrin on PALM2-mediated ESCC cell migration, ezrin knockout KYSE450 cells were constructed using CRISPR/Cas9 technology, and two monoclonal cell lines were successfully obtained (Fig. 6*A*). Flag-PALM2 was transfected into both control and knockout cell lines (Fig. 6*B*). In a wound healing assay, PALM2 enhanced the migration of KYSE450 cells but not ezrin-knockout KYSE450 cells (Fig. 6*C*), suggesting that PALM2 enhanced the migration of ESCC through ezrin, and that ezrin is a downstream effector molecule of PALM2. Furthermore, the distribution of ezrin in the insoluble fraction and the plasma membrane was increased after overexpression of PALM2 (Fig. 6, *D* and *E*), and phosphorylation of ezrin Y146 was enhanced as well (Fig. 6*F*). This Y146 phosphorylation, located in the N-terminal FERM domain, is another indication of ezrin activation outside of the T567 site, the most familiar marker of ezrin activation (44). These results show that PALM2 contributes to ezrin activation and enhances the migration of ESCC cells.

## Discussion

PALM2 belonging to the paralemmin family is partially encoded by the fusion gene *PALM2AKAP2*. The family consisting of paralemmin-1 (PALM), PALM2, paralemmin-3 (PALM3), and palmdelphin (PALMD) are a poorly studied class of proteins. In this study, we show that the PALM2 CAAX motif at the C-terminus could be prenylated by FTase. Prenylated PALM2 interacts with ezrin at the K253/254/262/263 lysine residues in the ezrin FERM domain to activate ezrin and promote the migration of esophageal cancer cells. PALM2 is highly expressed in esophageal cancer tissues and is closely associated with poor prognosis of patients with esophageal cancer.

The earliest discovered member was PALM from which the family name is derived (45, 46). PALM is a protein implicated in plasma membrane dynamics, the development of filopodia, neurites, and dendritic spines, and the invasive and metastatic potential of cancer cells. PALM itself is a hydrophilic protein but has a hydrophobic tail due to prenylation with a CAAX structure at the C-terminus, enabling insertion into the plasma membrane (45). Here, we demonstrate that the FTase, FNTA, and FNTB interacts with and prenylated PALM2. The treatment with FTase inhibitor suppresses PALM2 membrane localization and reduces the stability of PALM2. Mutant PALM2^C408S^ does not localize to the plasma membrane, but was dispersed in the cytoplasm (Fig. 3). It is suggested the prenylation of PALM2 is catalyzed by FTase. Although proteins acquire lipid moieties after prenylation, their membrane localization will be affected if the CAAX motif lacks hydrolysis and carboxymethylation (47, 48). Almost all post-prenylation hydrolysis of AAX residues and carboxymethylation of the exposed cysteine in humans is accomplished by Ras-converting enzyme 1 (RCE1) and by isoprenylcysteine carboxyl methyltransferase (ICMT) in the endoplasmic reticulum (49). Many prenylated proteins are also palmitoylated in the Golgi apparatus to increase their lipophicity (50). PALM had been shown to be palmitoylated at C381 and C383 at its C-terminus (sequence: 381-CKCCSIM-387) (45). Coincidentally, the C-terminus of PALM2 (sequence: 405-CQCCVVM-411) is similar to PALM. Thus, prenylated PALM2 might undergo proteolysis, carboxymethylation and palmitoylation in sequence at the C-terminus, and then be targeted to the plasma membrane. Through online prediction and MS analysis, C270 was verified as a palmitoylation site in PALM2 (supplemental Fig. S3).

As a member of the ERM protein family, ezrin serves as an intermediate between the plasma membrane and the actin cytoskeleton. Activated ezrin regulates key events in a variety of cancers and interacts with different proteins to participate in different signaling pathways. Antagonistic relationships between ezrin and different Rho GTPases regulate cell migration and adhesion (51, 52). Ezrin binds to the GEF of Cdc42 to activate the downstream pathway of Cdc42 and promote the directed migration of breast cancer cells (53). Ezrin mediates cell growth and survival through Akt signaling in some cancers, which is essential for cancer proliferation, invasion, migration, and survival (33, 34, 54). Previous studies show that ezrin promotes the development of esophageal cancer by regulating the rearrangement of the cell cytoskeleton (27, 35, 36, 37). This study not only shows that PALM2 is upregulated in esophageal cancer and is an independent risk factor for poor prognosis in patients with esophageal cancer (Fig. 2) but also demonstrates that PALM2 activates ezrin (Fig. 6, *D*–*F*) and subsequently promotes the malignant progression of esophageal cancer cells (Fig. 6*C*). The conformational switch of ezrin from the dormant to the active form is complex (7). It is widely recognized that the interaction of lysine residues K253/254/262/263, in the ezrin FERM domain, with phosphatidylinositol, 4, 5-bisphosphate (PIP2) is important for activation (32, 38, 39). In this study, when lysines are mutated to asparagine at these sites, the interaction between ezrin and PALM2 is reduced (Fig. 5*F*). Upregulated PALM2 facilitates phosphorylation at ezrin Y146 and increases ezrin binding to plasma membrane. These results suggest that PALM2 anchors ezrin to the membrane.

Note that, although the predicted molecular weight of PALM2 from the UniProt database is about 45 kDa, Western blotting analysis from tissues and cell lines shows that PALM2 exists as 45 kDa and 75 kDa forms (Fig. 2, *D* and *E*). Unknown modifications of PALM2 might be responsible for its larger molecular weight. Therefore, we predicted the possible post-translational modifications online. Glycosylation and SUMOylation may result in large changes in molecular weight. We predicted PALM2 has the potential to be glycosylated based on O-glycosylation site prediction (https://services.healthtech.dtu.dk/services/YinOYang-1.2) and N-Glycosylation site prediction (https://services.healthtech.dtu.dk/services/NetNGlyc-1.0) (40) (supplemental Fig. S4, *A* and *B*). PALM2 has four potential sites of SUMOylation and two SUMO interaction regions (https://sumo.biocuckoo.cn/) (supplemental Fig. S4*C*). PALM2 also has many predicted sites of phosphorylation (https://services.healthtech.dtu.dk/services/NetPhos-3.1/) (supplemental Fig. S4*D*) (41), ubiquitination (http://gpsuber.biocuckoo.cn/), and acetylation (http://pail.biocuckoo.org/). In our previous proteomics data (22), we detected seven phosphorylated sites (S260, S262, S371, S376, S378, S382, S383) predicted online. The above and other modifications may be the reason for two PALM2 molecular weights. However, we cannot make a definite conclusion. In the future, we will explore the map of PALM2’s post-translational modifications.

In conclusion, we found that prenylated PALM2 can promote the migration of ESCC cells by recruiting ezrin to the plasma membrane and activating ezrin. Inhibiting the enzymes in the three steps of prenylation can block the prenylation of proteins, which affects the function of downstream interacting proteins and hinders the malignant progression of tumors in turn, but the mechanism of action between prenylated protein and interacting protein needs to be extensively explored in order to find the most accurate target on the “working line” and find a more precise point for the prevention and treatment of tumors and other diseases.

## Data Availability

The data that support the findings of this study was downloaded from PRIDE database (accession number PXD021701) or iProX database (accession number IPX0002501000). The mass spectrometry data of this study data has been deposited to the ProteomeXchange Consortium (http://proteomecentral.proteomexchange.org) *via* the iProX partner repository with the data set identifier PXD040969 (for identification of PALM2) and PXD040700 (for interacting proteins of PALM2).

## Supplemental data

This article contains supplemental data (22).

## Conflict of interest

The authors declare no competing interests.
