# Supplementary material for: Prenylated PALM2 Promotes the Migration of Esophageal Squamous Cell Carcinoma Cells Through Activating Ezrin
Source: Mol Cell Proteomics. 2023 Jun 15;22(8):100593. doi: 10.1016/j.mcpro.2023.100593 (PMC10393820; doi:10.1016/j.mcpro.2023.100593)

SUPPLEMENTARY FIGURE LEGENDS

**Supplementary Fig. S1 Identification of PALM2 in 45 kDa and 75 kDa.** HEK 293T cells were transfected with Flag-PALM2. Pierce™ Anti-DYKDDDDK Magnetic Agarose was used for immunoprecipitation. IP samples were subjected to SDS-PAGE and then 1 mm × 1 mm gel s around 45 kDa and 75 kDa were cut off for a LC-MS/MS.

**Supplementary Fig. S2 Prenylation influences the stability of PALM2.** *A*, mRNA level change of PALM2 with the treatment of FTI 277. *B*, protein level change of PALM2 with the treatment of FTI 277, MG132 and CQ.

**Supplementary Fig. S3 PALM2 is palmitoylated.** *A*, Prediction of PALM2’s S-palmitoylation in http://csspalm.biocuckoo.org/. *B*, Mass spectrometry analysis of the palmitoylated PALM2 peptide in the 75 kDa PALM2 (amino acids 254-271; sequence TVIADGSLSHPKEHMLCK). C, PALM2 distribution after 2-BP treatment via analysis of detergent-soluble and -insoluble fractions.

**Supplementary Fig. S4 PALM2 has many potential post-translational modifications.** *A*, Prediction of PALM2 O-glycosylation in <https://services.healthtech.dtu.dk/services/YinOYang-1.2/>. *B*, Prediction of PALM2’s N-glycosylation in <https://services.healthtech.dtu.dk/services/NetNGlyc-1.0/>. *C*, Prediction of PALM2 SUMOylation in <https://sumo.biocuckoo.cn/>. *D*, Prediction of PALM2 phosphorylation in <https://services.healthtech.dtu.dk/services/NetPhos-3.1/>.

**Supplementary Fig. S5 Unprenylated PALM2 cannot interact with ezrin.** *A*, KYSE30 cells were stably transfected with Flag-PALM2 or PALM2^C408S^, and then Flag IP samples were probed by western blotting with antibodies against Flag and ezrin. *B*, HEK 293T cells were co-transfected with GFP-EZR and Flag-PALM2 or PALM2^ΔCAAX^, followed by Flag IP. Immunoprecipitated samples were probed by western blotting with antibodies against Flag and GFP.

**Supplementary Fig. S1**


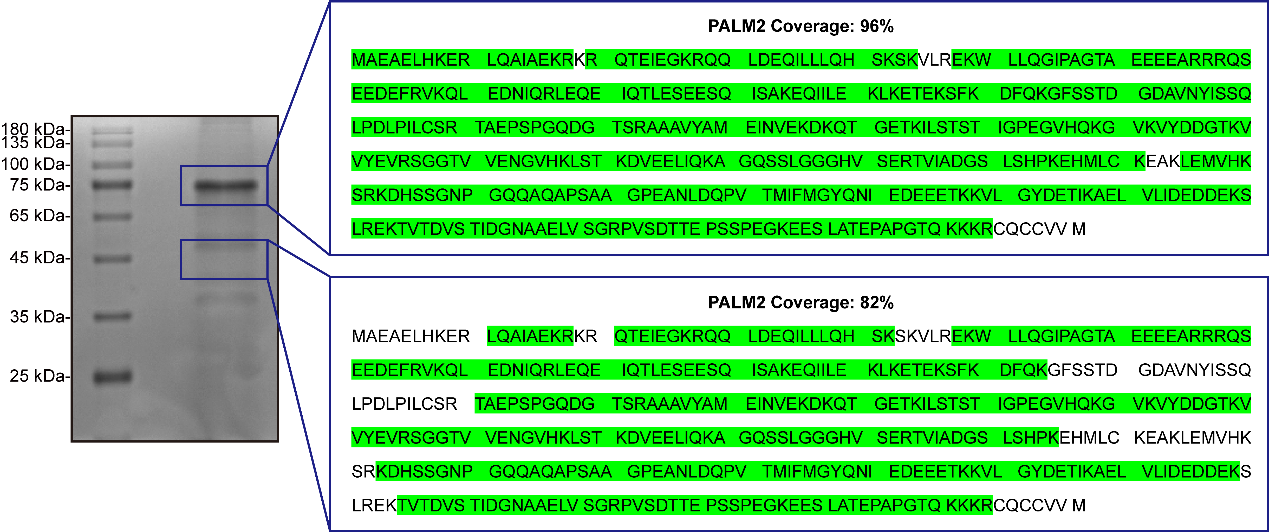


**Supplementary Fig. S2**


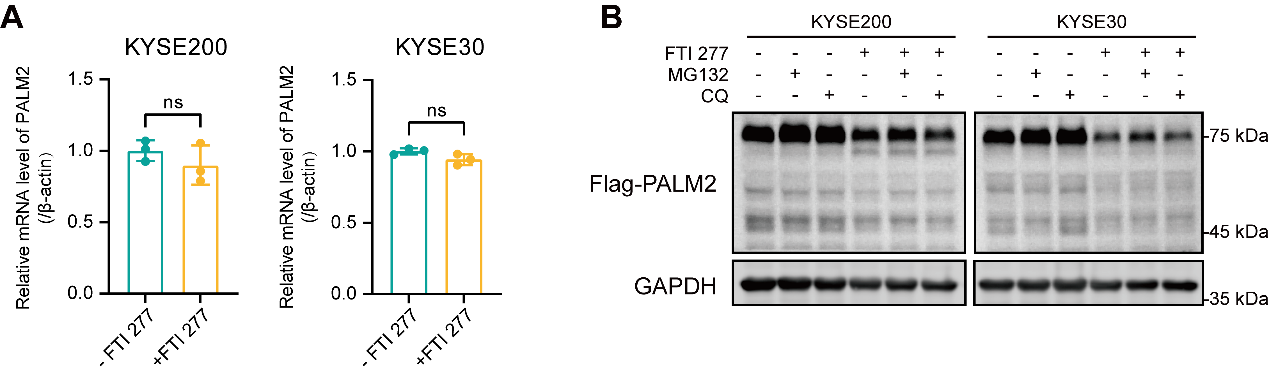


**Supplementary Fig. S3**


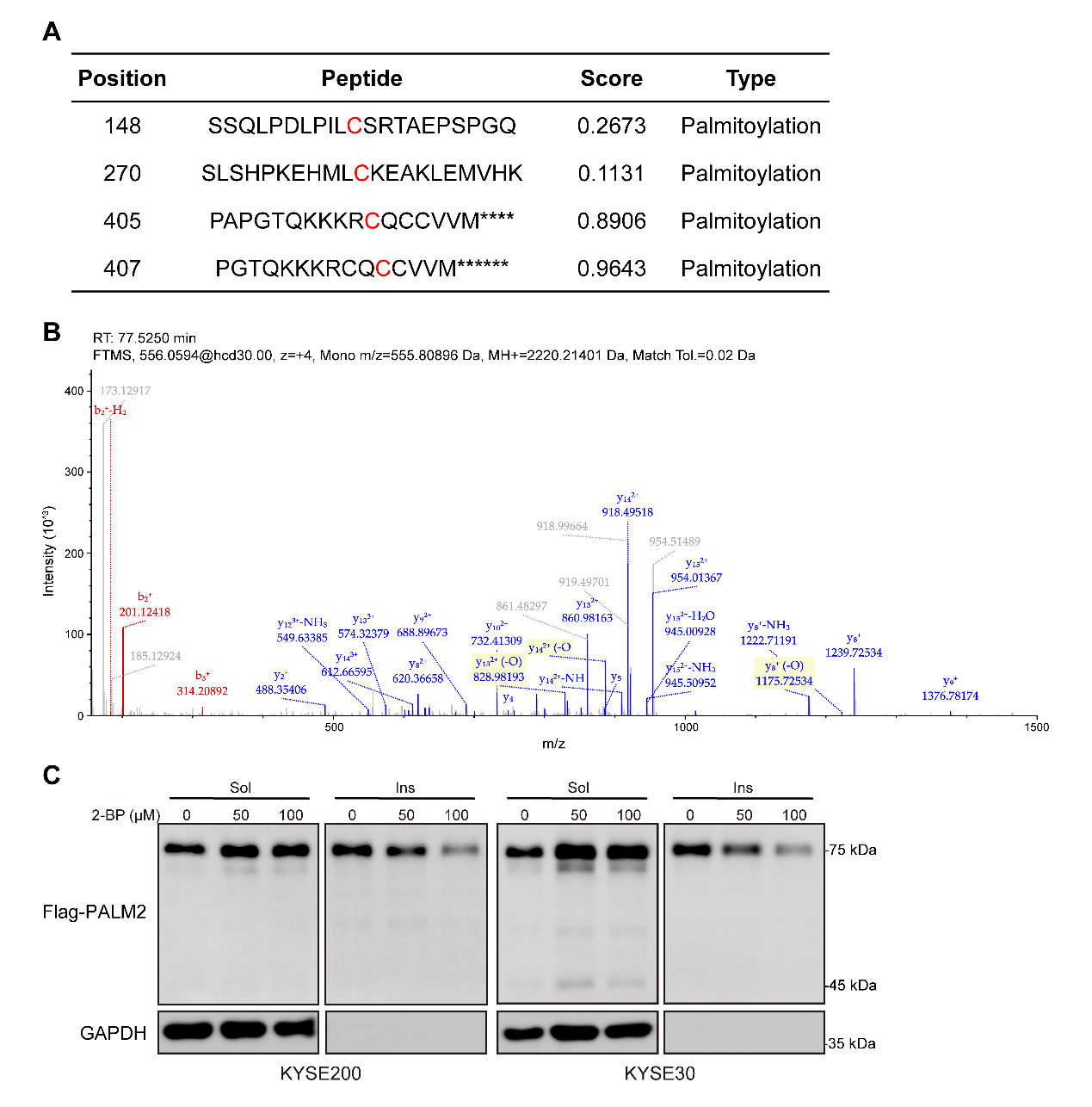


**Supplementary Fig. S4**


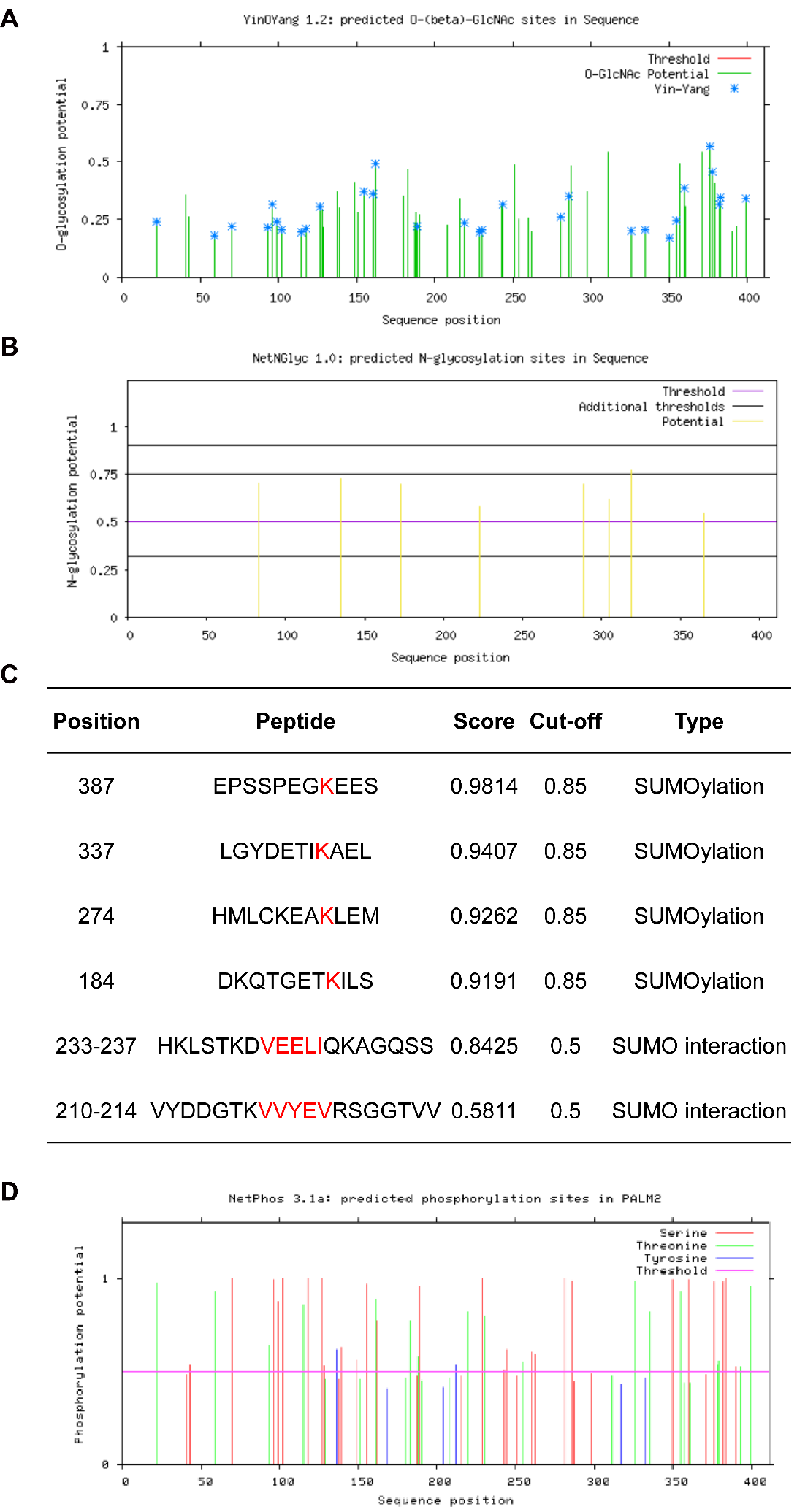


**Supplementary Fig. S5**


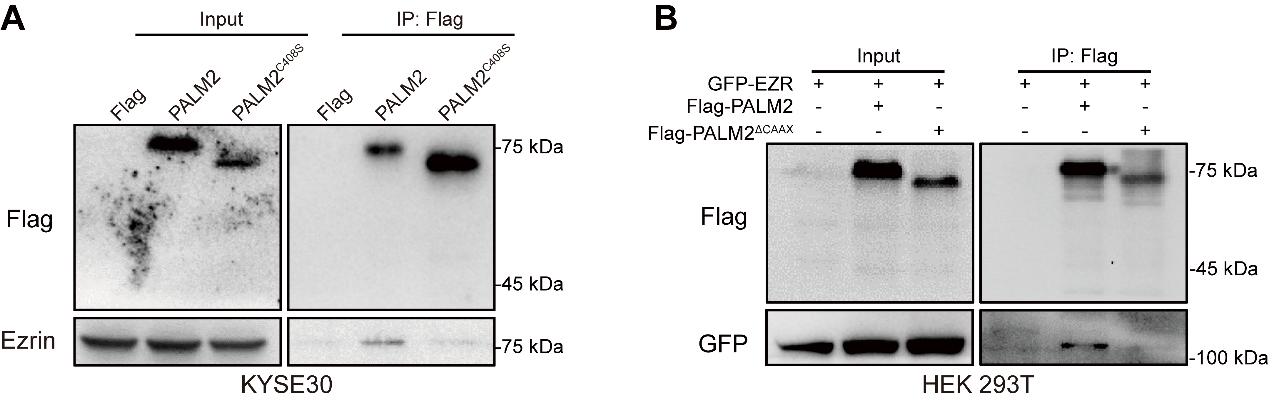

Supplement: Supplemental Figures S1–S5 [file mmc1.docx]
