# Supplementary material for: Prenylated PALM2 Promotes the Migration of Esophageal Squamous Cell Carcinoma Cells Through Activating Ezrin
Source: Mol Cell Proteomics. 2023 Jun 15;22(8):100593. doi: 10.1016/j.mcpro.2023.100593 (PMC10393820; doi:10.1016/j.mcpro.2023.100593)
Supplement: Supplemental Tables S2 and S3 [file mmc3.docx]

| **S****upplementary Table S2 The clinical and pathological characteristics of 124 patients with esophageal cancer in the proteomic data** | | | | | |
| --- | --- | --- | --- | --- | --- |
| **Clinical and pathological indexes** | **Case No.** | **5-year OS (%)** | ***P* value*** | **5-year DFS (%)** | ***P* value*** |
| Specimens | 124 |  |  |  |  |
| Mean age | 58.52 |  |  |  |  |
| Age (years) |  |  |  |  |  |
| ≤58 | 62 | 37.1 | 0.734 | 28.8 | 0.275 |
| >58 | 62 | 49.4 |  | 44.4 |  |
| Gender |  |  |  |  |  |
| Male | 97 | 43.3 | 0.962 | 37.0 | 0.934 |
| Female | 27 | 43.0 |  | 35.4 |  |
| Smoke |  |  |  |  |  |
| Yes | 42 | 33.3 | 0.252 | 33.3 | 0.474 |
| No | 82 | 48.6 |  | 38.3 |  |
| Drink |  |  |  |  |  |
| Yes | 14 | 14.3 | 0.019 | 14.3 | 0.008 |
| No | 110 | 47.0 |  | 39.5 |  |
| Tumor location |  |  |  |  |  |
| Upper | 6 | 33.3 | 0.959 | 33.3 | 0.935 |
| Middle | 75 | 46.4 |  | 37.9 |  |
| Lower | 43 | 39.5 |  | 34.9 |  |
| Histological grade |  |  |  |  |  |
| G1 | 23 | 52.2 | 0.400 | 47.8 | 0.072 |
| G2 | 82 | 43.3 |  | 37.0 |  |
| G3 | 19 | 31.6 |  | 21.1 |  |
| Primary tumor |  |  |  |  |  |
| T1+T2 | 20 | 54.2 | 0.256 | 54.5 | 0.170 |
| T3+T4 | 104 | 41.0 |  | 33.1 |  |
| Regional lymph node |  |  |  |  |  |
| N0 | 61 | 58.9 | 0.000 | 54.0 | 0.000 |
| N1 | 34 | 46.2 |  | 33.6 |  |
| N2 | 22 | 9.1 |  | 4.5 |  |
| N3 | 7 | 0 |  | 0 |  |
| pTNM stage |  |  |  |  |  |
| I+II | 61 | 58.9 | 0.001 | 54.0 | 0.000 |
| III+IV | 63 | 27.4 |  | 19.2 |  |
| *Log-rank test of Kaplan-Meier analysis; *P* < 0.05 was considered significant. OS, overall survival; DFS, disease free survival. | | | | | |

**Supplementary Table S3 List of primers**

| Name of plasmid | Primer sequence |
| --- | --- |
| pBOBi-N-3×Flag-PALM2 | F：agagaattcggatccgcagaggcggaattgcacaaggaaa  R：cttccatggctcgagtcacatgacaacacagcattgacag |
| pBOBi-N-3×Flag-PALM2^C408S^ | F：agagaattcggatccgcagaggcggaattgcacaaggaaa  R：cttccatggctcgagtcacatgacaacagagcattgacagcgc |
| pBOBi-N-3×Flag-PALM2^ΔCAAX^ | F：agagaattcggatccgcagaggcggaattgcacaaggaaa  R：cttccatggctcgagtcagcattgacagcgctttttcttttgg |
| pBOBi-C-3×HA-FNTB | F：agagaattcggatccgccaccatggcttctccgagttctttca  R：cttccatggctcgaggtcggttgcaggctctg |
| pBOBi-N-Myc-FNTA | F：agagaattcggatccgcggccaccgagggggt  R：cttccatggctcgagttattgctgtacatttgttggtgagtcattttctgtgctgt |
